# Supplementary figures and images for: Deep learning insights into the architecture of the mammalian egg-sperm fusion synapse
Source: eLife. 2024 Apr 26;13:RP93131. doi: 10.7554/eLife.93131 (PMC11052572; doi:10.7554/eLife.93131)

Figure 1, left

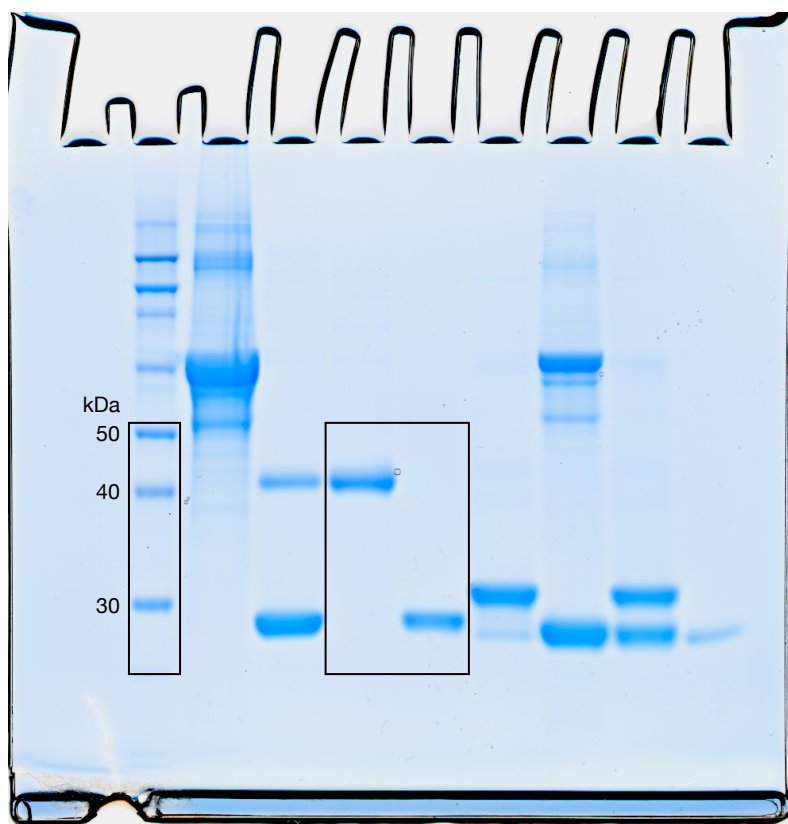

Figure 1, right

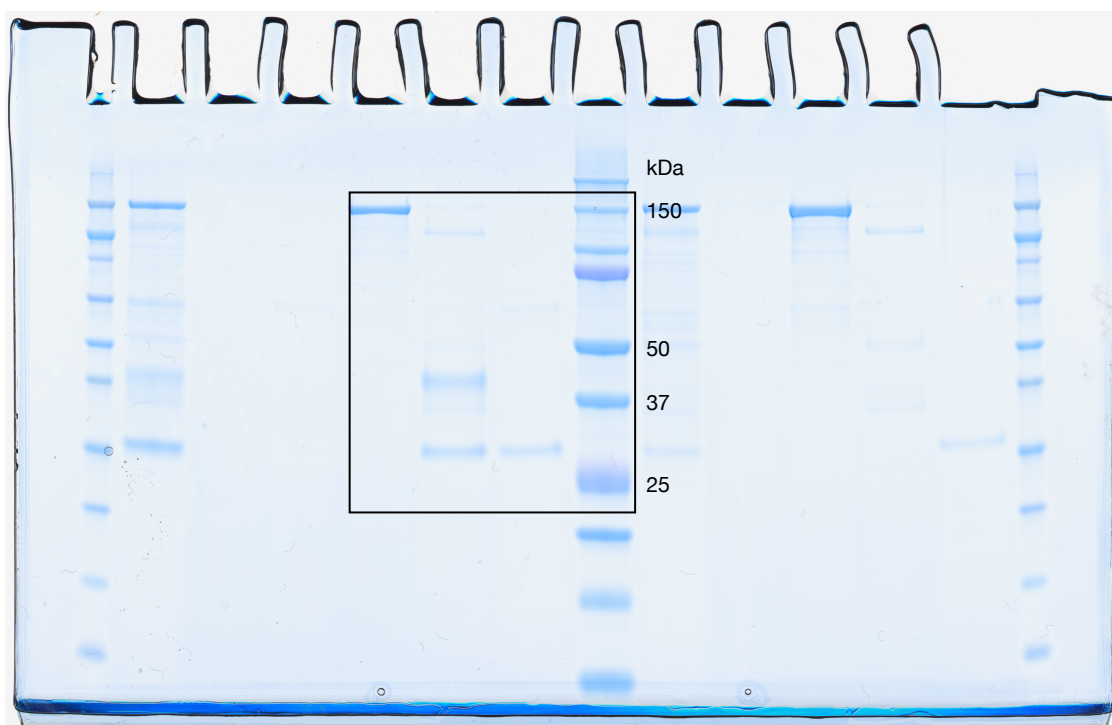

Supplement: Figure 1—source data 1. [file elife-93131-fig1-data1.zip › eLife-VOR-RA-2023-93131R1_figure_1-source_data/Figure 1ΓÇôSource Data Labelled.pdf]

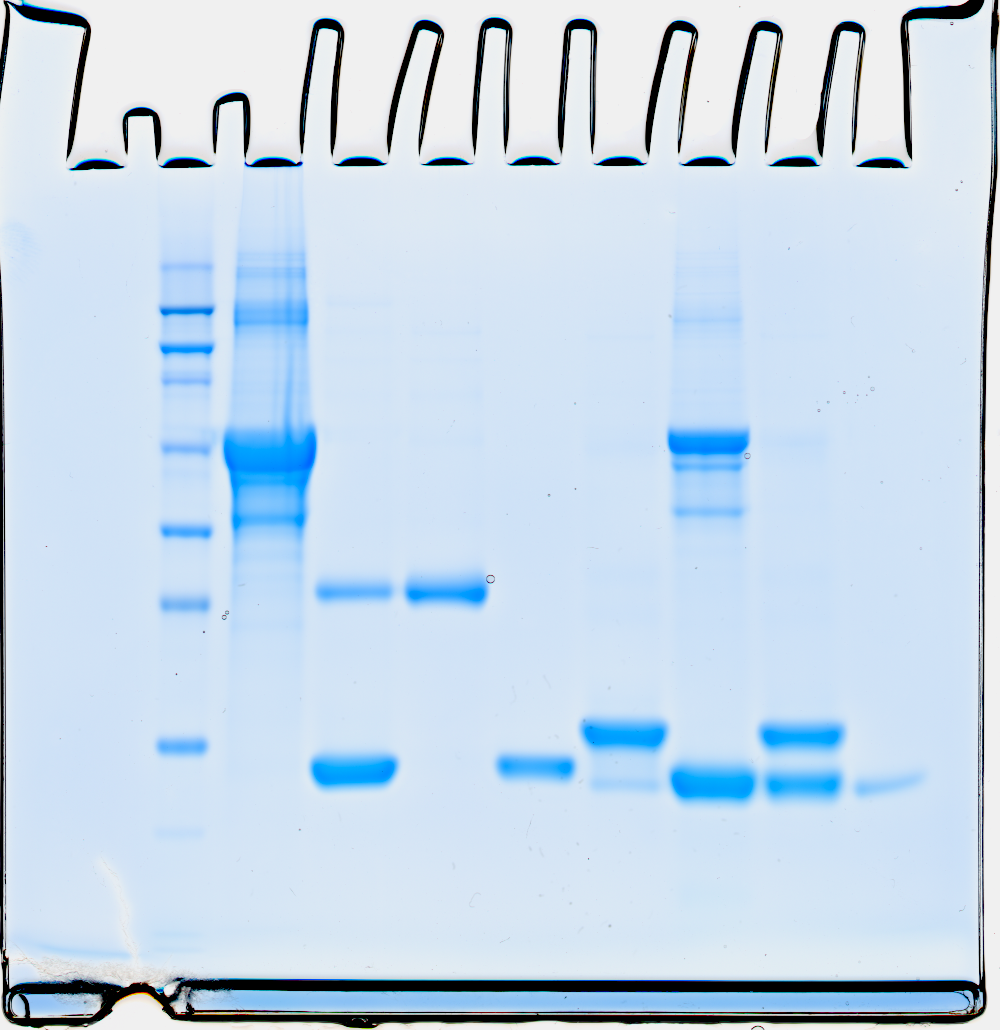

Supplement: Figure 1—source data 1. [file elife-93131-fig1-data1.zip › eLife-VOR-RA-2023-93131R1_figure_1-source_data/Figure 1ΓÇôSource Data 1.tif]

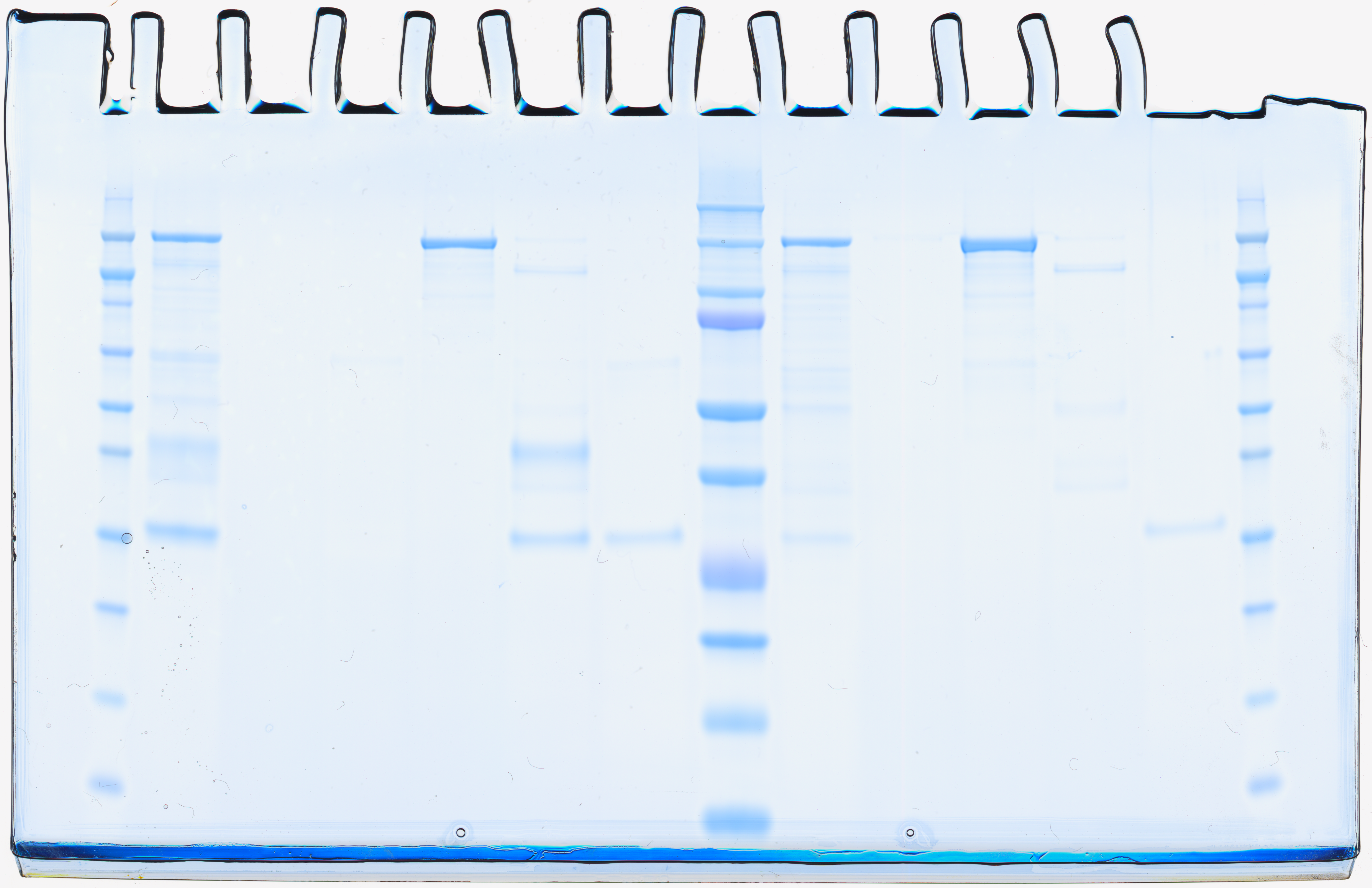

Supplement: Figure 1—source data 1. [file elife-93131-fig1-data1.zip › eLife-VOR-RA-2023-93131R1_figure_1-source_data/Figure 1ΓÇôSource Data 2.tif]

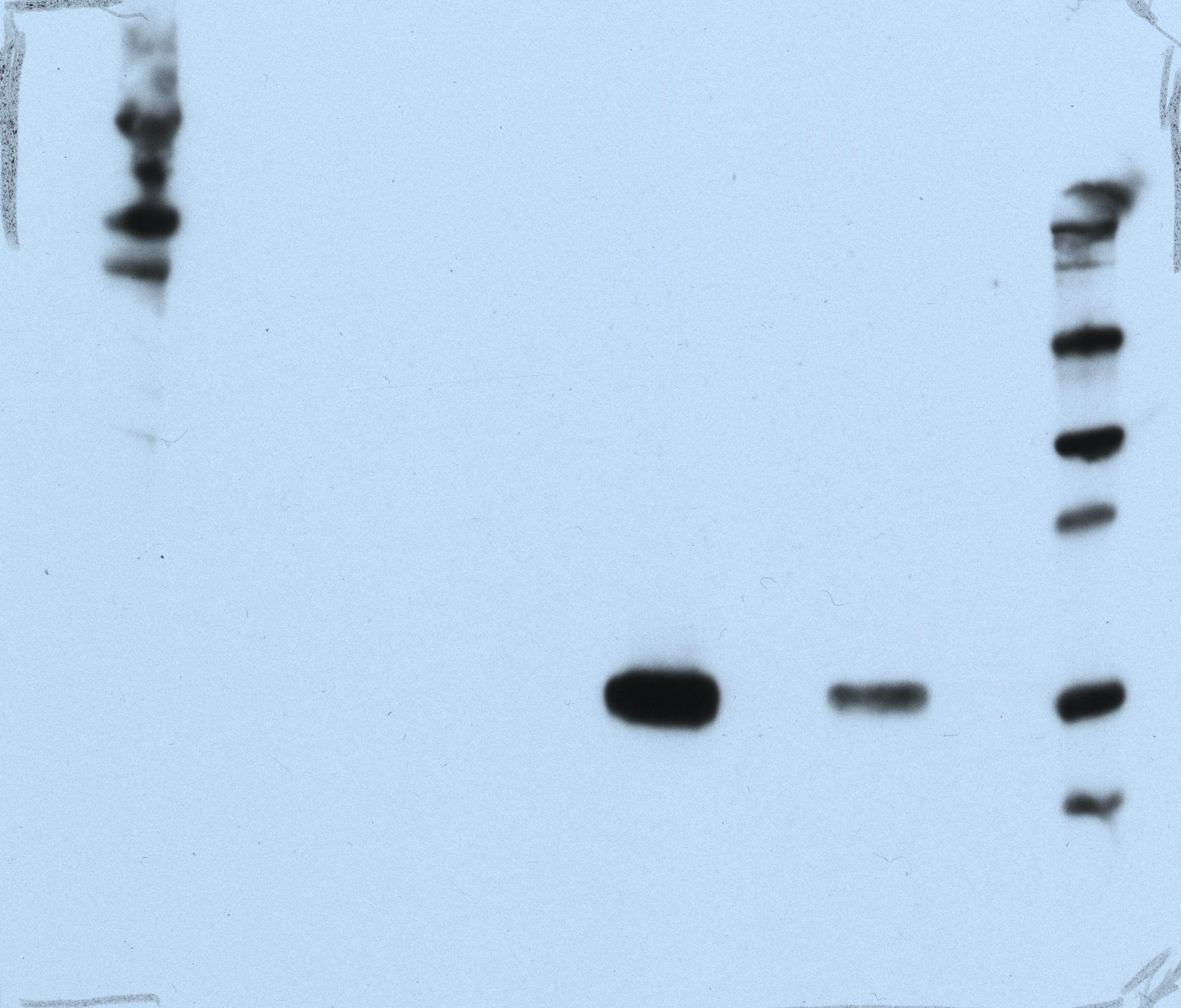

Supplement: Figure 1—figure supplement 1—source data 1. [file elife-93131-fig1-figsupp1-data1.zip › eLife-VOR-RA-2023-93131R1_figure_1-figure_supplement_1/Figure 1-Figure Supplement 1ΓÇôSource Data 1.tif]

**Figure 1-Figure Supplement 1, left**

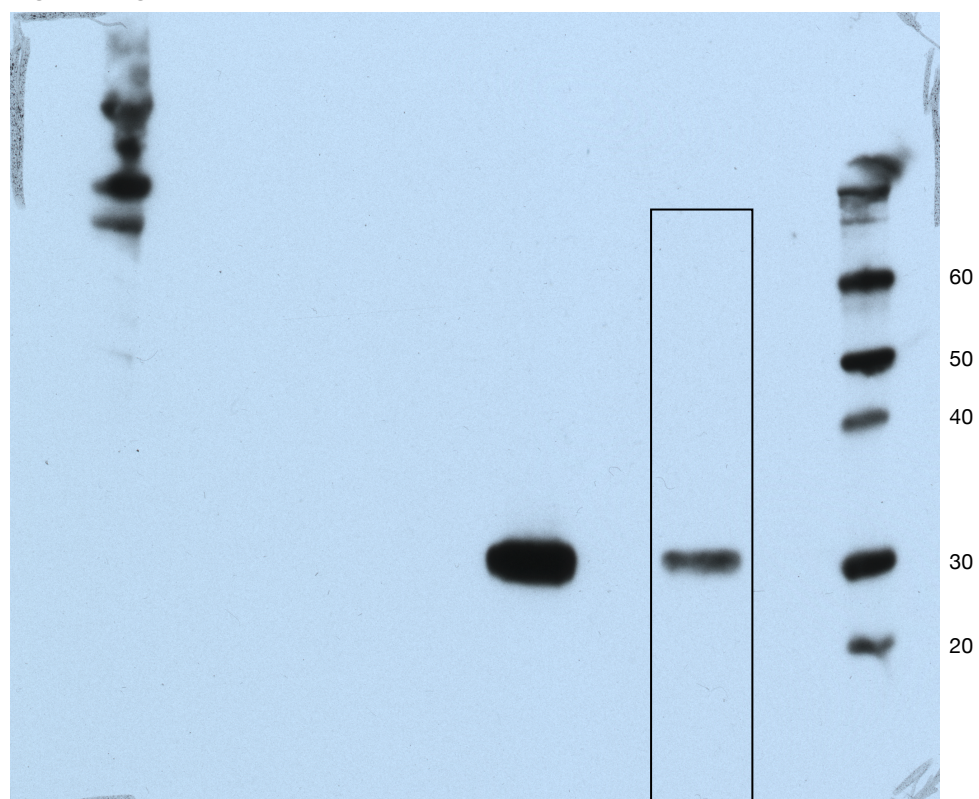

**Figure 1-Figure Supplement 1, right**

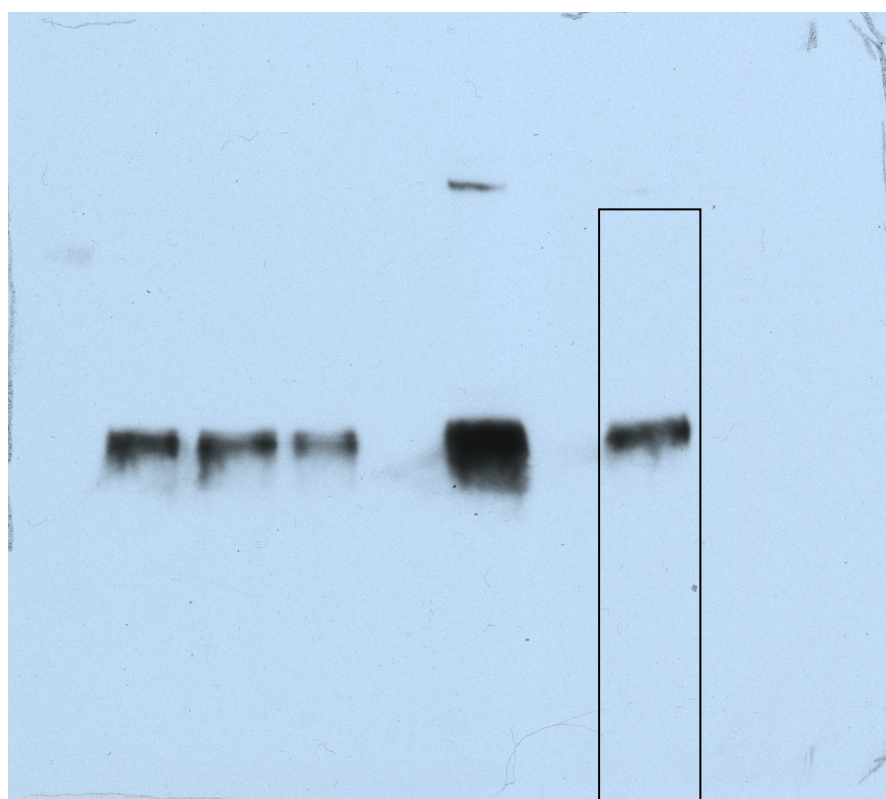

Supplement: Figure 1—figure supplement 1—source data 1. [file elife-93131-fig1-figsupp1-data1.zip › eLife-VOR-RA-2023-93131R1_figure_1-figure_supplement_1/Figure S1ΓÇôSource Data Labelled.pdf]

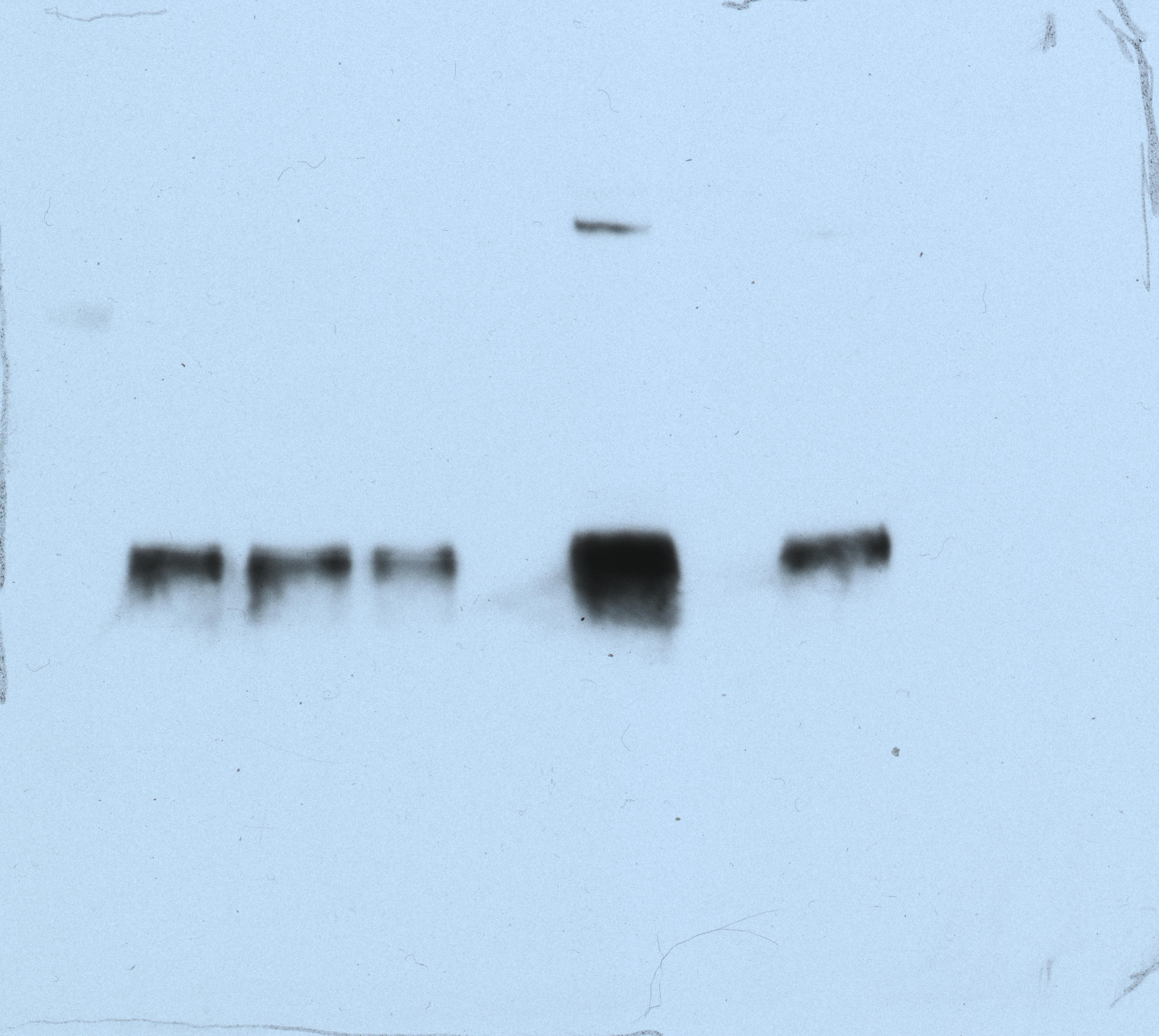

Supplement: Figure 1—figure supplement 1—source data 1. [file elife-93131-fig1-figsupp1-data1.zip › eLife-VOR-RA-2023-93131R1_figure_1-figure_supplement_1/Figure 1-Figure Supplement 1ΓÇôSource Data 2.tif]

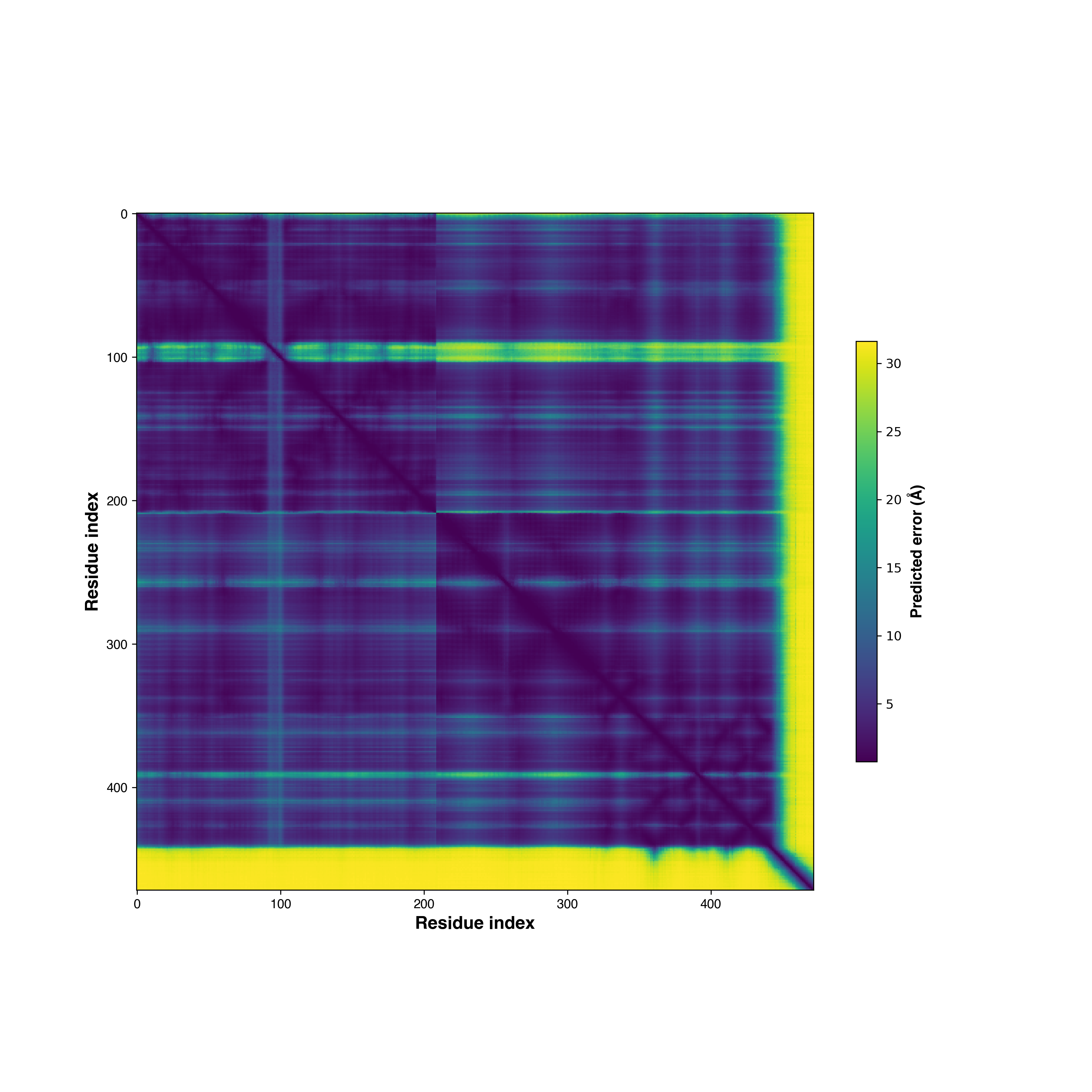

Supplement: Figure 2—source data 1. [file elife-93131-fig2-data1.zip › eLife-VOR-RA-2023-93131R1_figure_2-source_data/figure_2bd/hJUNO_hIZUMO1_ranked_0_PAE.png]

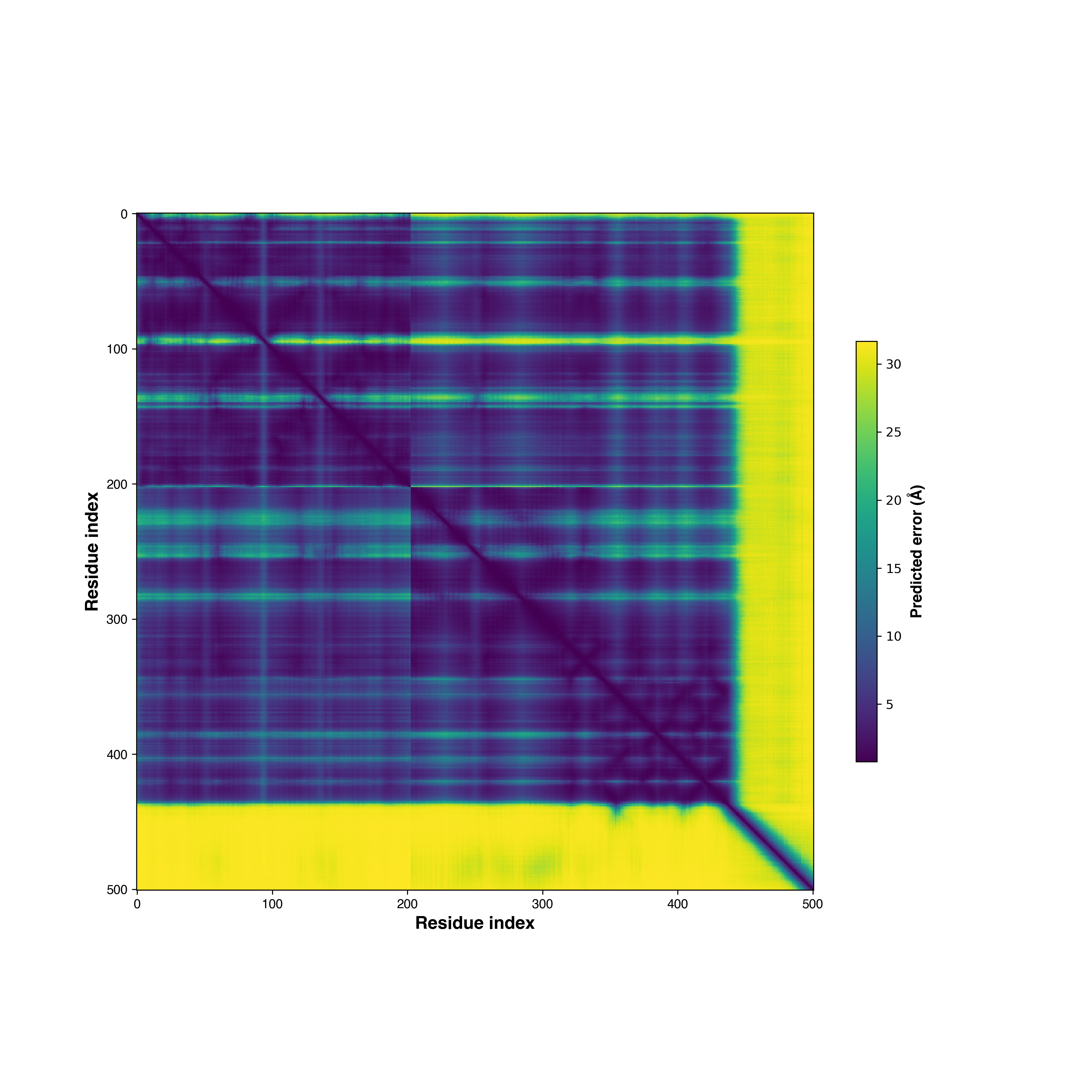

Supplement: Figure 2—source data 1. [file elife-93131-fig2-data1.zip › eLife-VOR-RA-2023-93131R1_figure_2-source_data/figure_2ce/mJUNO_mIZUMO1_ranked_0_PAE.png]
